# Nutritional Status and Post-Cardiac Surgery Outcomes: An Updated Review with Emphasis on Cognitive Function

**DOI:** 10.3390/jcm13144015

**Published:** 2024-07-09

**Authors:** Norsham Juliana, Nur Adilah Shuhada Abd Aziz, Sofwatul Mokhtarah Maluin, Noor Anisah Abu Yazit, Sahar Azmani, Suhaini Kadiman, Kamilah Muhammad Hafidz, Nur Islami Mohd Fahmi Teng, Srijit Das

**Affiliations:** 1 Faculty of Medicine and Health Science, Universiti Sains Islam Malaysia, Nilai 71800, Negeri Sembilan, Malaysia; sofwatulmal@usim.edu.my (S.M.M.); drazmanisahar@kpju.edu.my (S.A.); 2 Department of Anesthesia and Intensive Care, Institut Jantung Negara, Kuala Lumpur 50400, Malaysia; nradilahaziz@gmail.com (N.A.S.A.A.); anisahyazit@gmail.com (N.A.A.Y.); suhaini@ijn.com.my (S.K.); dr.kamilah@ijn.com.my (K.M.H.); 3 KPJ Research Centre, KPJ Healthcare University, Nilai 71800, Negeri Sembilan, Malaysia; 4 Faculty of Health Science, Universiti Teknologi MARA, Puncak Alam 40450, Selangor, Malaysia; nurislami@uitm.edu.my; 5 Department of Human & Clinical Anatomy, College of Medicine & Health Sciences, Sultan Qaboos University, Muscat 123, Oman; s.das@squ.edu.om

**Keywords:** malnutrition, cardiac surgery, pre-operative nutrition, post-operative cognitive dysfunction (POCD), inflammation

## Abstract

**Background/Objectives:** Nutritional status significantly influences cardiac surgery outcomes, with malnutrition contributing to poorer results and increased complications. This study addresses the critical gap in understanding by exploring the relationship between pre-operative nutritional status and post-operative cognitive dysfunction (POCD) in adult cardiac patients. **Methods**: A comprehensive search across key databases investigates the prevalence of malnutrition in pre-operative cardiac surgery patients, its effects, and its association with POCD. Factors exacerbating malnutrition, such as chronic illnesses and reduced functionality, are considered. The study also examines the incidence of POCD, its primary association with CABG procedures, and the impact of malnutrition on complications like inflammation, pulmonary and cardiac failure, and renal injury. **Discussions**: Findings reveal that 46.4% of pre-operative cardiac surgery patients experience malnutrition, linked to chronic illnesses and reduced functionality. Malnutrition significantly contributes to inflammation and complications, including POCD, with an incidence ranging from 15 to 50%. CABG procedures are particularly associated with POCD, and malnutrition prolongs intensive care stays while increasing vulnerability to surgical stress. **Conclusions**: The review underscores the crucial role of nutrition in recovery and advocates for a universally recognized nutrition assessment tool tailored to diverse cardiac surgery patients. Emphasizing pre-operative enhanced nutrition as a potential strategy to mitigate inflammation and improve cognitive function, the review highlights the need for integrating nutrition screening into clinical practice to optimize outcomes for high-risk cardiac surgery patients. However, to date, most data came from observational studies; hence, there is a need for future interventional studies to test the hypothesis that pre-operative enhanced nutrition can mitigate inflammation and improve cognitive function in this patient population.

## 1. Introduction

Nutritional status is significantly important in cardiac surgery in view of patients with (or at risk of) malnutrition suffering worse post-operative outcomes [1]. Malnutrition is related to a poor surgical outcome and to a higher prevalence of comorbidities and mortality, as a result of which the healing time is prolonged and the cost increases [2,3]. Additionally, malnutrition is an important factor that negatively affects mental and physical functions [4]. Coronary artery bypass grafting (CABG) surgical procedure is a major stress factor that can activate several inflammatory and catabolic pathways in patients. Consequently, post-operative cognitive decline (POCD), delirium, and dementia are commonly diagnosed among cardiac surgery patients. Nutritional status has been found to be one of the risk factors for the mentioned post-operative complications [5,6]. It is believed that an appropriate nutritional status allows the body to react appropriately to this stressor and recover faster and more efficiently. Lopez-Delgado et al. (2013) highlight strategies in prehabilitation programs that align with Enhanced Recovery After Surgery (ERAS) principles to optimize patients pre-surgery. These programs focus on nutritional support, physical activity, and metabolic enhancement using dietary modifications and supplements rich in antioxidants and omega-3 polyunsaturated fatty acids. Evidence indicates that these interventions improve metabolic parameters and reduce post-operative complications, such as atrial fibrillation, leading to shorter hospital stays. Additionally, promoting healthy dietary habits and providing nutritional counseling before cardiac surgery can reduce cardio-metabolic risk factors, optimizing patient readiness and enhancing surgical outcomes [1].

Moreover, sarcopenia and frailty, compounded by malnutrition, significantly impair cognitive outcomes in post-heart surgery patients. These conditions collectively diminish physical resilience and metabolic function critical for cognitive recovery. We focused on nutritional status due to its direct impact on post-operative recovery in cardiac surgery patients. Malnutrition affects vital processes like wound healing and immunity, crucial for recovery after surgery. Sarcopenia, marked by muscle loss and decreased strength, correlates with post-surgery cognitive decline [7]. Frailty, reflecting reduced physiological reserves and heightened vulnerability to stressors, exacerbates cognitive impairment in these patients [8]. The interrelated nature of these conditions highlights the necessity of cohesive nutritional interventions and comprehensive management strategies to enhance cognitive outcomes and overall recovery in this patient population.

Managing POCD involves a multifaceted approach that includes both preventive and therapeutic strategies. Pre-operative assessments should identify at-risk patients, with early interventions ensuring adequate caloric and protein intake. Specialized supplements like antioxidants and omega-3 fatty acids can help manage inflammation from cardiopulmonary bypass (CPB) [9]. Intraoperatively, minimizing surgical stress through advanced techniques and careful anesthesia management can also reduce the likelihood of cognitive decline. Post-operatively, early mobilization, cognitive therapy, and continued nutritional support are essential in managing POCD [10]. Additionally, close monitoring and early identification of cognitive impairments allow for timely interventions, improving overall recovery and reducing the long-term impact of POCD.

However, explanations related to the effect of poor nutritional status pre-operatively on the incidence of POCD, specifically after cardiac surgery, are still lacking. Therefore, this paper aims to review the current incidence of malnutrition among cardiac surgery patients and explore in depth the relationship between nutritional status pre-operatively and the incidence of POCD among this population.

## 2. Methods

A literature search was conducted using six databases, namely PubMed, Elsevier, ScienceDirect, Google Scholar, SpringerLink, and ClinicalKey. Publications in the English language were used for this review. The terms used to search the related articles were “malnutrition”, “post-operative cognitive decline”, “cardiac surgery”, “nutritional status”, “hospitalized patient”, “nutritional assessment”, and “cognitive dysfunction”. The full articles were obtained if their abstracts explained malnutrition, referring to undernutrition, factors, and effects of malnutrition pre-and post-operatively, and the incidence of POCD after surgery, focusing on adult cardiac surgery patients.

## 3. Prevalence of Heart Disease

The growing epidemic of cardiovascular disease (CVD) and the increasing number of aging populations globally have resulted in CVD remaining the number one cause of death for decades [11,12]. According to studies, CVD caused 422.7 million cases and 17.9 million deaths in 2015, and it may result in over 23.6 million deaths yearly by 2030 [13,14,15]. Recent data from the American Heart Association’s (AHA) Heart Disease and Stroke Statistics—2023 Update provide a comprehensive view of coronary heart disease (CHD) prevalence among US adults. According to the National Health and Nutrition Examination Survey (NHANES) 2017–2020, the overall CHD prevalence is 7.1%, with males at 8.7% and females at 5.8%. Ethnic variations from the National Health Interview Survey (NHIS) 2018 show rates of 5.7% among White adults, 5.4% among Black adults, 8.6% among American Indian/Alaska Native adults, and 4.4% among Asian adults [16].

Heart disease is the primary cause of mortality for men, women, and members of the majority of racial and ethnic groups in the United States. It is reported that a person dies due to CVD every 33 s in the United States [17]. In the United States, heart disease caused approximately 695,000 deaths in 2021, which is one death out of every five [16]. As a result, between 2018 and 2019, heart disease cost the United States $239.9 billion a year [17]. This covers the expense of medical treatment, medications, and lost wages as a result of mortality [17].

Over the past three decades, CVD mortality and morbidity in Malaysia have increased. According to the Malaysian Ministry of Health Report, CVD has remained the leading cause of death since 1980 [18]. In 2020, 13.7% of 109,164 medically certified deaths were caused by ischemic heart disease (IHD), an increase of 2.0% from the previous year [19]. However, the percentage of medically certified deaths due to IHD decreased to 13.7% in 2021 in view of the world experiencing the COVID-19 pandemic, including Malaysia. This has made COVID-19 infection the leading cause of death in Malaysia for 2021 (19.8%), followed by IHD [20].

## 4. Trends and Outcomes of Cardiac Surgery

Generally, most large datasets were collected prior to the COVID-19 pandemic. The number of procedures performed in developed countries such as the United States (US) and the United Kingdom (UK) has been relatively constant over the past two decades. Despite the fact that there is an increase in the mean patient age, comorbidity burden, and logistic EuroSCORE, the outcomes have consistently improved [21]. Data before the pandemic showed that cardiac operative mortality had fallen between 2.1% and 2.2% in developed countries such as the UK and the US [22,23]. However, the global strike of the COVID-19 pandemic has unprecedentedly affected healthcare management, including decisions on performing surgical procedures for cardiac patients. The stark reduction in the inpatient provision of cardiovascular procedures was seen worldwide during the two years. Regardless of socioeconomic status, the pandemic is associated with greater risk-adjusted mortality (odds ratio 1.398) [24].

Nationwide data in Malaysia did not specifically address post cardiac surgery’s mortality and morbidity rate. The best referral is from the National Heart Institute (IJN) annual report. A similar trend of improvement was reported over the recent decade until the pandemic struck. The in-hospital mortality rate for patients undergoing isolated CABG in IJN was consistently low, ranging from 3.7% in 2016 to 3.0% in 2018 [25]. Recently, IJN also reported a higher actual survival rate for all open-heart surgery (94.4%) and isolated CABG (96.1%) in 2022 as compared to the predicted survival rate estimated by the Parsonnet scoring system (predicted survival rate ranging from 91.7% to 92.2%) [26]. Cardiac surgery is seen to be more difficult in patients with complicated medical histories. At the time of surgery, the majority of the patients had hypertension (74.7%) and hypercholesterolemia (74.8%), and nearly half of them had diabetes mellitus (47.4%) in 2022 [26]. The improvement indicates that the nation’s main referral center for cardiac cases, including cardiac surgery, has advanced at par with the developed countries. Since the mid-1980s, operative mortality has been included as part of the indicator for the quality of healthcare in developed countries. Consequently, the US Society of Thoracic Surgeons (STS) formed the Adult Cardiac Surgery Database (ACSD) in 1989 as a formal report on the outcomes of cardiac procedures. The 30-day mortality has been an important metric guiding the evolution of the cardiac operative approach [22]. Nevertheless, a direct comparison of the trend between countries may not be relevant as the contributing factors to the provision of surgical procedures for cardiac patients differ.

### 4.1. Malnutrition among Cardiac Surgery Patients

The American Society for Parenteral and Enteral Nutrition (ASPEN) defined malnutrition as “An acute, subacute, or chronic state of nutrition in which varying degrees of overnutrition or undernutrition with or without inflammatory activity have led to a change in body composition and diminished function” [27]. Malnourished patients undergoing cardiac surgery have been shown to have worse post-operative outcomes, including higher morbidity and mortality [9,28].

#### 4.1.1. Pre-Operative Malnutrition: The Possible Cause

Pre-operative hospitalized patients had a high rate of malnutrition, reaching up to 46.4% among cardiac surgery patients [29]. Acute and chronic diseases among cardiac surgery patients cause changes in body composition, resulting in poor nutritional status among this population [30]. In addition to having poor nutrition uptake or intake, these patients frequently have impaired neurophysiological status or physical mobility [31,32]. As a result of their illness, patients with heart failure are usually not physically active, which causes the disease to worsen. A longer stay in the hospital prior to surgery also results in less physical activity [33]. Hospitalized patients tend to experience high levels of anxiety and depression, which may also negatively impact their nutritional intake before surgery [34]. Sarcopenia and physical performance are impacted by malnutrition due to changes in the nervous and skeletal systems as well as loss of muscle mass and strength [35,36,37]. All three have negative health effects, including increased morbidity and mortality; and decreased quality of life [35,38,39,40]. Thus, all these factors might cause further nutritional state deterioration among pre-operatively hospitalized patients. Additionally, patients with malnutrition frequently have decreased cardiac muscle mass, which eventually lowers cardiac output and reduces renal perfusion. Furthermore, low levels of energy, micronutrients, and electrolytes alter cytokines, glucocorticoids, insulin, and insulin-like growth factors, all of which affect cardiac function [29]. Congestive heart failure further decreases appetite and food intake, leading to the pathophysiological circulus vitiosus [29,41].

Appetite changes among heart failure patients are common. A study conducted by Andrea et al. (2021) demonstrated that decreased appetite is a common issue for HF patients, both right before and 1.5 years after hospitalization [42]. It is reported that almost 38% of heart failure patients had an appetite level at risk of future weight loss, based on the Council on Nutrition Appetite Questionnaire (CNAQ) assessment [43]. The condition of the disease itself may impact the patients’ appetite. Heart failure may cause fluid retention and accumulation, bowel edema, and shortness of breath, making it difficult for patients to eat [43]. Most patients with heart failure are elderly, and the process of aging itself may impact appetite hormones negatively. A study carried out by Andrea et al. (2021) found that almost half of the patients (49%) were discharged with decreased appetite [42]. The condition worsened when further appetite decreases were seen among 141 patients (22%) after 18 months of follow-up. Another study conducted by Pilgrim et al. (2016) also found a consistent decrease in the patient’s appetite over time [44]. Comorbidity, which includes signs of depression, sleep disorders, and impaired cognitive function, is a common issue in heart failure. It has been demonstrated to have a negative effect on appetite in general populations, but the evidence is limited in heart failure populations. Physical activity is a crucial part of managing heart failure, but it is unclear how it affects appetite. Additionally, medical management of heart failure is essential for symptom relief and lowering neurohormonal activation, but it may also have adverse effects on food intake. Many heart failure patients also experience loneliness, which could also affect their appetite [42]. Furthermore, decreased appetite over time was independently predicted by fatigue, depressive symptoms, and poor quality of life. This problem occurs persistently and cannot be solved spontaneously. Hence, a patient’s appetite should be evaluated daily, similarly to other crucial nutritional evaluations such as weight. Thus, early nutritional counselling could be provided to avert or delay malnutrition [42].

Numerous studies have also demonstrated that immunological and neurohormonal disorders are key contributors to cardiac cachexia [45]. The development of cardiac cachexia depends on comprehensive overexpression of catabolic mechanisms. This is due to the numerous immunologic, metabolic, and neurohormonal alterations [46,47,48,49]. Patients with heart failure have increased energy expenditure and concurrently decreased calorie intake. As a result, compensatory proteolysis occurs, therefore causing muscle wasting. These two processes are frequently linked to the development of cachexia [49,50,51]. Hypoalbuminemia is also one of the pathogenic stages that follow in the development of cardiac cachexia. A significant proteolysis causes nitrogen balance among heart failure patients. It appears that hypoalbuminemia causes changes in the activity of inflammatory cytokines, resulting in an increase in the secretion of TNF-alpha. TNF-alpha is a tumor necrosis factor which is one of the main inflammatory cytokines of the human body. TNF-alpha has an impact on the endothelium, negatively causing the tissue blood flow to be lower, and subsequently reducing the absorption of nutrients and physical fitness [49,50,51]. Other than TNF-alpha, IL-1 and IL-6 are also secreted, and these can lead to the activation of catabolic processes [45,47,49]. Additionally, hypoalbuminemia causes a reduction in the transforming growth factor activity (anti-inflammatory cytokines IL-10 and TGF-beta) [49,52,53]. Thus, it can be concluded that hypoalbuminemia and muscle wasting are interrelated. However, the specific reason and order of the previously mentioned changes are still unclear.

Additionally, hormonal changes are common among heart failure patients. Lower testosterone and an increase in cortisol blood concentration led to an increase in catabolic processes, adding to the proteolysis and muscle wasting [53]. Elevated catabolic processes cause a decrease in testosterone levels and an increase in blood concentration of cortisol, adding to muscle wasting and proteolysis. Reduced body weight induces adiponectin secretion, promoting lipid oxidation and insulin sensitivity. As a result, concentrations of blood lipid and glucose become lower. Besides that, weight loss also can cause leptin concentration to increase. Leptin activity is similar to adiponectin, whereby it increases the sensitivity towards insulin and reduces lipogenesis [49,53,54]. Nonetheless, heart failure patients commonly have insulin resistance [55]. Additionally, leptin is mainly responsible for activating the satiety center, whereby it promotes the feeling of early satiety and lack of nutrient intake [56]. This eventually will aggravate cardiac cachexia.

High energy expenditure in cardiac cachexia affects the balance between the sympathetic and parasympathetic systems. It increases the secretion of cortisol and renin–aldosterone–angiotensin system (RAAs) activation and natriuretic peptide system [49,50,51]. Angiotensin II plays a major role in cachexia. It initiates proteolysis in the muscular tissue by elevating the secretion of IL-6 and glucocorticosteroids and reduces the IGF-1 secretion [50]. Some researchers demonstrated that the muscle fibril proteins are mostly degraded in neoplastic cachexia via the ubiquitin-dependent mechanism of protein degradation [49,57]. The processes are similar in cardiac cachexia. Their increased activity is brought on by angiotensin II-induced nicotinamide adenine dinucleotide phosphate (NADPH) activation, which results in increased oxidative stress in muscle tissue [49,50,51]. The activation of muscular satellite cells is reduced by angiotensin II, which interferes with the repair of damaged muscle fibers. Additionally, it speeds up the breakdown of lysosome and proteasome proteins [50], triggers the corticoid-releasing hormone (CRH) secretion, and lowers the neuropeptide Y (NPY) and orexin secretion [51]. As a result, it stimulates a feeling of satiety and lowers the appetite.

Altered psychosocial and lifestyle further worsened the malnutritional status among this group. Ringaitienė et al. Ringaitienė, Gineitytė [29] reported that psychological stress and neuropsychological issues, such as dementia and depression, contributed to the incidence of malnutrition. This shows that there is a strong relationship between these conditions, and it is important to practice a proper psychological screening for the patients before surgical procedure. In general, Lomivorotov et al. [28] and Stoppe et al. [9] stated that cardiac surgery patients with malnutrition can have poor outcomes. This includes deterioration of chronic heart failure, cardiac cachexia and sarcopenia, less metabolic and functional reserves, increased hemodilution (including coagulopathy, need for transfusion, and inflammation), and gastrointestinal endothelial dysfunction. Table 1 below summarizes the multifactorial aspects and consequences of malnutrition in cardiac surgery patients.

#### 4.1.2. Effect of Malnutrition Post-Operatively

Nutritional deficits result in the reduction in defense mechanism and reduced metabolic reserves leading to the pathomechanism of inflammation in cardiac patients [58]. Cardiac surgery patients commonly experience complex systemic inflammatory response syndrome. This can be seen through the manifestation of tachycardia, pyrexia, leukocytosis, edema, hypotension, and organ failure post-operatively [58]. Additionally, the most common organ malfunction after heart surgery is pulmonary failure (up to 79%) [59], ventricular failure (up to 70%) [60,61], poor post-operative cognitive function (up to 65%) [62,63], and acute kidney injury in a third of the patients. Particularly, high-risk cardiac patients undergoing prolonged cardiac surgeries and cardiopulmonary bypass (CPB) are more susceptible to an elevated inflammatory response with adverse effects.

Organ malfunction leads to a longer stay in the intensive care unit (ICU) and a prolonged requirement for life-sustaining treatments. The patients’ health deteriorates even more when malnutrition led to the onset of endothelial gut mucosa malfunction, permitting the translocation of potential bacterial [64]. Patients who are malnourished are therefore more prone to surgical stress, ischemia-and-reperfusion damage, complications from anesthesia, hemodilution, and inflammation. Despite this, pre-operative fasting and delayed post-operative nutritional supplementation are frequently reported, aggravating the already-existing malnutrition. Numerous observational studies have shown severe macro- and micronutrient depletion as well as the significance of energy and protein metabolism in the initial stages following heart surgery [9,28].

Longer hospital stays are common among malnourished patients. Higher percentage of hospitalization longer than 7 days among malnourished cardiac surgical patients was reported in a study by Ávila et al. [65]. A study by Karst et al. [66] which evaluated 83 cardiac patients in ICU showed that the mean hospital stay of malnourished patients was longer than well-nourished patients. While these cohort and observational studies suggest a potential link between nutritional status and cognitive outcomes, they may not be able to provide direct evidence for the efficacy of pre-operative nutritional interventions. A recent review by Dong et al. [67] has shown associations between pre-operative nutritional markers and post-operative cognitive function in cardiac surgery patients, but interventional studies are lacking. A small pilot study by Jones et al. (2024) explored pre-operative nutritional supplementation in cardiac surgery patients, showing promising results for cognitive outcomes, but larger randomized controlled trials are needed to establish causality.

Another retrospective analysis study carried out by Drover et al. [68] also demonstrated that post-surgical patients were more likely to become malnourished while in the post-operative ICU. Malnutrition can cause adverse effects on the post-operative outcome. This includes poor functional recovery, high risk of infection, increased incidence of pneumonia, and prolonged length of stay in ICU, which leads to a lower discharge-to-home rate and increased morbidity and mortality [69]. In this case, nutritional support might be the best intervention to prevent further deterioration of nutritional status among this group.

According to ESPEN guidelines [70], nutritional support has been shown to help in improving the recovery process in many ways. This includes maintaining the metabolism, maintaining the gut function, reducing post-operative complications, promoting wound healing, and maintaining hydration and euglycemia adequacy. Good nutritional status with adequate dietary intake may also have a good impact on brain function. The processes that carry energy from foods to neurons are probably essential for regulating brain activity [69]. Energy intakes regulate cerebral oxygenation, making it a crucial component for the preservation of brain function by providing energy to the brain tissue, and this includes cerebral microcirculation [69].

Recent studies underscore the critical role of nutritional status in predicting outcomes following cardiac surgery. Almohammadi et al. [71] highlighted that lower levels of the prognostic nutritional index (PNI) in heart surgery patients correlate with prolonged hospital stays and increased morbidity and mortality rates. Their study involving 264 individuals emphasized the significance of PNI and other nutritional markers in assessing risk in this patient population, despite mortality differences not reaching statistical significance across PNI levels. Similarly, Kawanishi et al. [72] investigated the impact of pre-operative malnutrition on disability-free survival after elective cardiac and thoracic aortic surgery in older patients. Their findings indicated that poor pre-operative nutritional status significantly reduced the likelihood of disability-free survival at one year post-surgery, reinforcing the critical need for nutritional optimization strategies in pre-operative care protocols for improved surgical outcomes.

#### 4.1.3. Association of Malnutrition with Cognitive Function Post-Operatively

After cardiac surgery, including aortic valve replacement (AVR) and coronary artery bypass grafting (CABG), post-operative cognitive dysfunction (POCD) is a common complication. Between 15% and 50% of individuals undergoing cardiac surgery have POCD [73]. According to a retrospective study, the most frequent cause of POCD following a cardiac operation is a CABG procedure, with incidence rates of 37.6% in the first week and 20.8% in the third month [74].

Predisposing factors include older age, female gender, higher bleeding episodes, and elevated post-operative creatinine levels [74]. Laalou et al. [75] reported that the incidence of POCD is influenced by age and the timing of assessment, with rates of 23–29% in patients aged 60–69 and over 70 years one week post-surgery, decreasing to 14% in those over 70 years by the third month post-surgery. Recent advances highlight the importance of considering nutritional status as a potential modifiable factor influencing cognitive outcomes in this population [76]. This comprehensive approach enables the identification and targeted intervention of specific cognitive deficits.

Additionally, numerous studies revealed that the primary factor contributing to the development of POCD is the inflammatory reactions linked to surgery [77,78,79,80]. It has been reported that inhibiting this inflammatory response can enhance cognitive recovery from surgery. Before surgery, enteral administration of enriched nutrition was thought to be able to trigger the vagal anti-inflammatory reflex, which would restrict the inflammatory response and subsequently decrease POCD [81,82]. This mechanism also can be seen in a study by Hovens et al. [83], where enteral enriched nutrition before surgery in young rats was found able to reduce systemic inflammation and improve cognitive performance after surgery. However, old rats showed a mixed favorable/unfavorable cognitive response without effect on systemic inflammation.

## *5.* Nutritional Screening and Assessment

Nutritional screening and assessment among cardiac patients are crucial aspects of comprehensive care, as they help identify individuals who may be at risk for poor nutritional status and allow healthcare providers to implement appropriate interventions. Poor nutritional status among cardiac patients has been associated with worse outcomes in a pre-operative setting [65,84]. In addition, malnutrition is prevalent among critical and high-risk cardiac patients, making it vital to identify those who may be malnourished or at increased risk of malnutrition [84]. Varieties of parameters used in the nutritional assessment include anthropometric data, clinical history, biochemical parameters, physical examinations, and dietary records [65]. Several nutritional screening tools have been proposed and evaluated for cardiac surgery patients. Lomivorotov et al. [85] conducted a comparative study to evaluate the prognostic value of nutritional screening tools for patients scheduled for cardiac surgery and found that nutritional screening tools can effectively identify patients at high risk of malnutrition [85]. Another study in agreement with the previous outcome also highlighted the importance of implementing nutritional screening in clinical routine practice to identify patients at nutritional risk and initiate optimization strategies [86].

Subjective Global Assessment (SGA) has been widely used in assessing the nutritional status of the hospitalized patient. SGA is practical and sensitive in identifying patients with impaired nutritional status. The subjective assessment that combines both clinical and dietary information allows patients to be classified into three categories: well-nourished, moderately malnourished, or severely malnourished [65,84]. Previous study proves that SGA is one of the tools that can effectively identify patients at high risk of malnutrition. In the evaluation, SGA includes weight loss, changes in subcutaneous fat, muscle wasting, and the presence of edema in its clinical factors. In addition to the clinical factors, the dietary factors included are changes in appetite, dietary intake, and gastrointestinal symptoms [87]. Due to the fact that all these factors are subjective, its data are highly dependent on the operator. Therefore, multiple operators may result in different variants of results.

In addition to SGA, there are also many widely used nutritional screening and assessment tools including the Mini Nutritional Assessment Short-Form (MNA-SF), Malnutrition Screening Tool (MST), Malnutrition Universal Screening Tool (MUST), and Nutrition Risk Screening 2002 (NRS-2002). However, currently there is still no validated nutritional screening and assessment tool developed specifically for this population. Additionally, the nutritional screening and assessment tool was rarely used in cardiac surgery [58]. Lomivorotov et al. [85] found that most nutrition screening tools are insufficiently sensitive to the risk of developing post-operative complications [28]. Identifying the nutritional status of hospitalized cardiac patients is essential to determine the most appropriate dietary treatment and to optimize health professionals’ and institutional managers’ planning [65].

Another screening tool that has the potential for screening nutritional status among post-cardiac surgery patients is the Controlling Nutritional Status (CONUT). The tool combines several objective parameters, and it is simple to calculate in order to obtain the nutritional status score. The score was obtained from blood albumin level, serum total cholesterol, and total lymphocyte count. The score ranges from 0 to 12, whereby higher scores indicate greater nutritional risk [88]. The multifaceted objective parameters taken into account allow a more nuanced evaluation among sensitive patients such as post-cardiac surgery patients as their nutritional status is susceptible to both acute and chronic aspects such as inflammation, lipid metabolism, and protein synthesis. Furthermore, this tool has the potential to detect subtle nutritional changes that can go unnoticed with the marker assessments. Thus, prompt interventions can be conducted to improve patients’ conditions. However, further validations are needed in order to include CONUT as the standard nutritional assessment tool for post-surgery patients [89]. A list of nutritional assessment tools is presented in Table 2.

As highlighted, it is clear that nutritional assessment is also part of important assessment upon admission. In the event that malnutrition is identified, interventions should include pre-operative nutrition optimization, potentially delaying elective surgery to implement intensive nutritional support, and providing oral nutritional supplements enriched with immunonutrients [9,90]. Perioperative nutrition support should prioritize early enteral nutrition (EN) within 24–48 h post-surgery when feasible [91]. In cases where EN is contraindicated or insufficient, parenteral nutrition (PN) should be initiated within 3–7 days post-surgery, depending on the severity of malnutrition and clinical status.

The timing of perioperative PN requires careful consideration. For severely malnourished patients, PN may be initiated 7–10 days before surgery if adequate oral or enteral intake cannot be achieved [90]. In the immediate post-operative period, PN should be reserved for patients unable to meet >60% of their energy and protein requirements through EN within 3–7 days after surgery [92]. The decision to initiate PN should be made on a case-by-case basis, considering the patient’s nutritional status, expected duration of inadequate oral/enteral intake, and potential risks. By implementing these interventions and carefully timing nutritional support, we believe that the negative impact of malnutrition on cognitive outcomes and overall recovery in post-heart surgery patients can be mitigated, though further research is needed to establish optimal protocols specific to cardiac surgery patients and evaluate long-term effects on cognitive function.

## 6. Conclusions and Future Recommendations

Nutrition is one of the fundamental components listed in the ERAS protocols. Due to the fact that all open-heart surgeries are high-risk in nature, diligent consideration of adequate nutrition ensures optimum recovery and mitigates potential adverse complications [1]. Despite significant advancements in cardiac surgery techniques and post-operative care, there remains a critical gap in our understanding of the long-term cognitive outcomes and their nutritional correlates in post-heart surgery patients. While ERAS protocols have shown promise in improving various aspects of patient recovery, their impact on cognitive function and the potential role of nutrition in this context are not yet fully elucidated. Recent studies have suggested a possible link between perioperative nutritional status and post-operative cognitive decline, but the specific mechanisms and optimal nutritional interventions remain unclear [67]. Further research is needed to explore the complex interplay between nutritional factors, ERAS implementation, and cognitive outcomes in this patient population, with a particular focus on identifying modifiable risk factors and developing targeted interventions to enhance both short-term recovery and long-term cognitive health [92].

Managing malnutrition in cardiac surgery patients presents a complex challenge influenced by both chronic cardiovascular conditions and acute perioperative demands. While malnutrition often stems from physiological effects such as reduced appetite and metabolic dysregulation, addressing pre-operative nutritional deficits is crucial to enhance patient readiness for surgery. This approach complements the treatment of underlying diseases by bolstering physiological reserves and potentially reducing surgical complication. Thus, an accurate standardized assessment of nutritional status among this pool of patients pre- and post-operatively is crucial. To date, there is a paucity of universally recognized gold-standard nutrition assessment tools tailored for cardiac surgery patients. This patient cohort has a diverse spectrum of functional states [93]. Therefore, it is imperative that any nutritional status assessment tool utilized be expeditious and objective and minimize the susceptibility to operator judgement. In a nutshell, the current endeavor is focused on identifying the most effective nutritional assessment tool. The integration of nutrition screening into clinical practice and the subsequent initiation of tailored nutritional support holds promising potential in optimizing cardiac patient outcomes.

## Figures and Tables

**Table 1:** Key findings of Malnutrition in Cardiac Surgery Patients.

Key Findings	Summary
Causes of Pre-operative Malnutrition	Acute and chronic diseases, impaired neurophysiological status, physical inactivity, anxiety, depression, sarcopenia, and decreased appetite due to heart failure and aging.
Impact on Post-operative Outcomes	Higher morbidity and mortality, decreased quality of life, and increased risks of complications such as cardiac cachexia and sarcopenia.
Pathophysiological Mechanisms	Includes hormonal changes (e.g., cortisol, testosterone), inflammatory cytokines (e.g., TNF-alpha, IL-6), hypoalbuminemia, and altered cardiovascular and metabolic functions.
Psychosocial Factors	Psychological stress, neuropsychological issues (e.g., depression, dementia), and lifestyle factors contribute to worsened nutritional status.

**Table 2:** Tools for nutritional status screening and their benefits, parameters, and limitations.

Tool	Characteristics/Parameters
Malnutrition Screening Tool (MST)	Benefits: Simple, quick, valid, incorporates key parameters (recent weight loss, recent poor dietary intake, BMI), and reliable Parameters: BMI and appetite Limitations: Issues with its sensitivity and specificity that lead to false negatives and false positives, are not suitable for populations such as athletes or individuals with specific health conditions that affect BMI. Population: Hospitalized, outpatient, and institutionalized adult patient
Malnutrition Universal Screening Tool (MUST)	Benefits: Simple, quick, valid, incorporates key parameters (BMI, unintentional weight loss, and the effect of acute disease on nutritional intake), able to risk stratify, clinically relevant as it takes into account current nutritional status and historical changes in weight, and suitable for in clinical settings or community. Parameters: BMI, weight loss, and estimates of the effect of illness on dietary intake are needed. Limitations: For screening of adult patients only, dependent on BMI. Population: ESPEN recommends its use limit at the community level.
Nutritional Risk Screening 2002 (NRS 2002)	Benefits: It assesses a wide range of parameters related to nutritional status (weight loss, BMI, food intake, gastrointestinal symptoms, and disease severity) and takes into account both the severity and impact of disease on the patient’s nutritional status. NRS 2002 is based on clinical evidence. Parameters: Weight loss (within the last 3–6 months), BMI, intake, severity of disease. Limitations: The complex scoring requires operator training, assessment on the disease severity is subjective, limited to hospitalized patients. Population: ESPEN recommended this tool for nutritional screening to identify risks in severely ill patients.
Subjective Global Assessment (SGA)	Benefits: Combines clinical judgement and patient history, incorporates multiple factors, includes qualitative and quantitative evaluation, suitable for a wide range of patients, and gold-standard measurement accepted in clinical settings. Parameters: Clinical history (weight loss history, dietary intake changes, gastrointestinal symptoms persisting for more than 2 weeks, and functional capacity) and physical examination (subcutaneous fat, muscle wasting, ankle and sacral edema, and ascites) Limitations: Operator-dependent (judgement and interpretation may vary from one operator to another), time-consuming, typically requires face-to-face evaluation. Population: ASPEN recommended this tool for gold-standard nutritional screening in hospitalized patient.
Mini Nutritional Assessment Short-Form (MNA-SF)	Benefits: Quick and simple assessment that is easy to administer, enables tracking over time as it takes into account status within the past 3 months. Parameters: BMI, lifestyle and mobility, dietary intake (number of meals per day, food and fluid intake), self-perception of health and nutrition, subjective assessment of unintentional weight loss, disease state and stress during the last 3 months. Limitations: Dependence on clinical judgement Population: Only for the geriatric population.
Controlling Nutritional Status (CONUT)	Benefits: Quick, comprehensive assessment of multiple objective parameters, quantitative, not operator-dependent, useful in diverse population, and cost-effective. Parameters: Serum albumin levels, total lymphocyte count, and total cholesterol Limitations: Other parameters of intake are not included, provides the nutritional status at the time the biomarkers are taken. Population: All inpatients

## Data Availability

No new data were created or analyzed in this study. Data sharing is not applicable to this article.

## References

[1] Lopez-Delgado J.C., Muñoz-del Rio G., Flordelís-Lasierra J.L., Putzu A. (2019). Nutrition in Adult Cardiac Surgery: Preoperative Evaluation, Management in the Postoperative Period, and Clinical Implications for Outcomes. J. Cardiothorac. Vasc. Anesth..

[2] Leiva Badosa E., Badia Tahull M., Virgili Casas N., Elguezabal Sangrador G., Faz Méndez C., Herrero Meseguer I., Izquierdo González À., López Urdiales R., Oca Burguete F.J., Tubau Molas M. (2017). Hospital malnutrition screening at admission: Malnutrition increases mortality and length of stay. Nutr. Hosp..

[3] Meier R., Stratton R. (2008). Basic concepts in nutrition: Epidemiology of malnutrition. E-Spen Eur. E-J. Clin. Nutr. Metab..

[4] Smith P.J., Blumenthal J.A. (2016). Dietary Factors and Cognitive Decline. J. Prev. Alzheimers Dis..

[5] Ringaitienė D., Gineitytė D., Vicka V., Žvirblis T., Šipylaitė J., Irnius A., Ivaškevičius J., Kačergius T. (2015). Impact of malnutrition on postoperative delirium development after on pump coronary artery bypass grafting. J. Cardiothorac. Surg..

[6] Fiorda-Diaz J., Nicoleta S., Bergese S.D., Prabhakar H. (2017). Chapter 7—Anesthetic Agents: Neurotoxics or Neuroprotectives. Essentials of Neuroanesthesia.

[7] Ansaripour A., Arjomandi Rad A., Koulouroudias M., Angouras D., Athanasiou T., Kourliouros A. (2023). Sarcopenia Adversely Affects Outcomes following Cardiac Surgery: A Systematic Review and Meta-Analysis. J. Clin. Med..

[8] Meece L.E., Yu J., Winchester D.E., Petersen M., Jeng E.I., Al-Ani M.A., Parker A.M., Vilaro J.R., Aranda J.M., Ahmed M.M. (2023). Prognostic Value of Frailty for Heart Failure Patients Undergoing Left Ventricular Assist Device Implantation: A Systematic Review. Cardiol. Rev..

[9] Stoppe C., Goetzenich A., Whitman G., Ohkuma R., Brown T., Hatzakorzian R., Kristof A., Meybohm P., Mechanick J., Evans A. (2017). Role of nutrition support in adult cardiac surgery: A consensus statement from an International Multidisciplinary Expert Group on Nutrition in Cardiac Surgery. Crit. Care.

[10] Kotekar N., Shenkar A., Nagaraj R. (2018). Postoperative cognitive dysfunction—Current preventive strategies. Clin. Interv. Aging.

[11] Stenling A., Häggström C., Norberg M., Norström F. (2020). Lifetime risk predictions for cardiovascular diseases: Competing risks analyses on a population-based cohort in Sweden. Atherosclerosis.

[12] Vandersmissen G.J.M., Schouteden M., Verbeek C., Bulterys S., Godderis L. (2020). Prevalence of high cardiovascular risk by economic sector. Int. Arch. Occup. Environ. Health.

[13] Borhanuddin B., Mohd Nawi A., Shah S.A., Abdullah N., Syed Zakaria S.Z., Kamaruddin M.A., Velu C.S., Ismail N., Abdullah M.S., Ahmad Kamat S. (2018). 10-Year Cardiovascular Disease Risk Estimation Based on Lipid Profile-Based and BMI-Based Framingham Risk Scores across Multiple Sociodemographic Characteristics: The Malaysian Cohort Project. Sci. World J..

[14] Negesa L.B., Magarey J., Rasmussen P., Hendriks J.M.L. (2020). Patients’ knowledge on cardiovascular risk factors and associated lifestyle behaviour in Ethiopia in 2018: A cross-sectional study. PLoS ONE.

[15] Ahmed A.A.A., Al-Shami A.M., Jamshed S., Zawiah M., Elnaem M.H., Mohamed Ibrahim M.I. (2020). Awareness of the Risk Factors for Heart Attack Among the General Public in Pahang, Malaysia: A Cross-Sectional Study. Risk Manag. Healthc. Policy.

[16] Tsao C.W., Aday A.W., Almarzooq Z.I., Anderson C.A.M., Arora P., Avery C.L., Baker-Smith C.M., Beaton A.Z., Boehme A.K., Buxton A.E. (2023). Heart Disease and Stroke Statistics—2023 Update: A Report From the American Heart Association. Circulation.

[17] CDC (2023). Heart Disease Facts.

[18] Noor Hassim I., Norazman M.R., Diana M., Khairul Hazdi Y., Rosnah I. (2016). Cardiovascular risk assessment between urban and rural population in Malaysia. Med. J. Malays..

[19] Malaysia M.O.H., Putrajaya M. (2016). Prevention of Cardiovascular Disease in Women.

[20] Khaw W.F., Chan Y.M., Nasaruddin N.H., Alias N., Tan L., Ganapathy S.S. (2023). Malaysian burden of disease: Years of life lost due to premature deaths. BMC Public Health.

[21] Grant S.W., Kendall S., Goodwin A.T., Cooper G., Trivedi U., Page R., Jenkins D.P. (2021). Trends and outcomes for cardiac surgery in the United Kingdom from 2002 to 2016. JTCVS Open.

[22] Chan P.G., Seese L., Aranda-Michel E., Sultan I., Gleason T.G., Wang Y., Thoma F., Kilic A. (2021). Operative mortality in adult cardiac surgery: Is the currently utilized definition justified?. J. Thorac. Dis..

[23] Sanders J., Cooper J., Mythen M.G., Montgomery H.E. (2017). Predictors of total morbidity burden on days 3, 5 and 8 after cardiac surgery. Perioper. Med..

[24] Kaplan E.F., Strobel R.J., Young A.M., Wisniewski A.M., Ahmad R.M., Mehaffey J.H., Hawkins R.B., Yarboro L.T., Quader M., Teman N.R. (2023). Cardiac Surgery Outcomes During the COVID-19 Pandemic Worsened Across All Socioeconomic Statuses. Ann. Thorac. Surg..

[25] Negara I.J. (2018). Annual Report 2017–2018.

[26] Petra M., Taib M., Ramli M., Jaafar N., Gaafar I. (2020). Performance of EuroSCORE and EuroSCORE II in Institut Jantung Negara (IJN), Kuala Lumpur, Malaysia. Sch. J. Appl. Med. Sci..

[27] Abd Aziz N.A.S., Teng N., Abdul Hamid M.R., Ismail N.H. (2017). Assessing the nutritional status of hospitalized elderly. Clin. Interv. Aging.

[28] Lomivorotov V.V., Efremov S.M., Boboshko V.A., Nikolaev D.A., Vedernikov P.E., Deryagin M.N., Lomivorotov V.N., Karaskov A.M. (2013). Prognostic value of nutritional screening tools for patients scheduled for cardiac surgery †. Interact. CardioVascular Thorac. Surg..

[29] Ringaitienė D., Gineitytė D., Vicka V., Žvirblis T., Šipylaitė J., Irnius A., Ivaškevičius J. (2016). Preoperative risk factors of malnutrition for cardiac surgery patients. Acta Medica Litu..

[30] Slee A., Birch D., Stokoe D. (2015). A comparison of the malnutrition screening tools, MUST, MNA and bioelectrical impedance assessment in frail older hospital patients. Clin. Nutr..

[31] Girish M., Bhattad S., Ughade S., Mujawar N., Gaikwad K. (2014). Physical activity as a clinical tool in the assessment of malnutrition. Indian Pediatr..

[32] Barker L.A., Gout B.S., Crowe T.C. (2011). Hospital Malnutrition: Prevalence, Identification and Impact on Patients and the Healthcare System. Int. J. Environ. Res. Public Health.

[33] Beveridge C., Knutson K., Spampinato L., Flores A., Meltzer D.O., Van Cauter E., Arora V.M. (2015). Daytime Physical Activity and Sleep in Hospitalized Older Adults: Association with Demographic Characteristics and Disease Severity. J. Am. Geriatr. Soc..

[34] Polikandrioti M., Goudevenos J., Michalis L.K., Koutelekos J., Kyristi H., Tzialas D., Elisaf M. (2015). Factors associated with depression and anxiety of hospitalized patients with heart failure. Hell. J. Cardiol..

[35] Kramer C.S., Groenendijk I., Beers S., Wijnen H.H., van de Rest O., de Groot L. (2022). The Association between Malnutrition and Physical Performance in Older Adults: A Systematic Review and Meta-Analysis of Observational Studies. Curr. Dev. Nutr..

[36] Tieland M., Trouwborst I., Clark B.C. (2018). Skeletal muscle performance and ageing. J. Cachexia Sarcopenia Muscle.

[37] Hébuterne X., Bermon S., Schneider S.M. (2001). Ageing and muscle: The effects of malnutrition, re-nutrition, and physical exercise. Curr. Opin. Clin. Nutr. Metab. Care.

[38] Guest J.F., Panca M., Baeyens J.-P., de Man F., Ljungqvist O., Pichard C., Wait S., Wilson L. (2011). Health economic impact of managing patients following a community-based diagnosis of malnutrition in the UK. Clin. Nutr..

[39] Correa-Pérez A., Abraha I., Cherubini A., Collinson A., Dardevet D., de Groot L., de van der Schueren M.A.E., Hebestreit A., Hickson M., Jaramillo-Hidalgo J. (2019). Efficacy of non-pharmacological interventions to treat malnutrition in older persons: A systematic review and meta-analysis. The SENATOR project ONTOP series and MaNuEL knowledge hub project. Ageing Res. Rev..

[40] Trombetti A., Reid K.F., Hars M., Herrmann F.R., Pasha E., Phillips E.M., Fielding R.A. (2016). Age-associated declines in muscle mass, strength, power, and physical performance: Impact on fear of falling and quality of life. Osteoporos. Int..

[41] Cicoira M., Anker S.D., Ronco C. (2011). Cardio-renal cachexia syndromes (CRCS): Pathophysiological foundations of a vicious pathological circle. J. Cachexia Sarcopenia Muscle.

[42] Andreae C., van der Wal M.H.L., van Veldhuisen D.J., Yang B., Strömberg A., Jaarsma T. (2021). Changes in Appetite During the Heart Failure Trajectory and Association with Fatigue, Depressive Symptoms, and Quality of Life. J. Cardiovasc. Nurs..

[43] Andreae C. (2018). Appetite in Patients with Heart Failure: Assessment, Prevalence and Related Factors.

[44] Pilgrim A.L., Baylis D., Jameson K.A., Cooper C., Sayer A.A., Robinson S.M., Roberts H.C. (2016). Measuring appetite with the simplified nutritional appetite questionnaire identifies hospitalised older people at risk of worse health outcomes. J. Nutr. Health Aging.

[45] von Haehling S., Schefold J.C., Lainscak M., Doehner W., Anker S.D. (2009). Inflammatory biomarkers in heart failure revisited: Much more than innocent bystanders. Heart Fail. Clin..

[46] Kalantar-Zadeh K., Anker S.D., Horwich T.B., Fonarow G.C. (2008). Nutritional and Anti-Inflammatory Interventions in Chronic Heart Failure. Am. J. Cardiol..

[47] Martins T., Vitorino R., Moreira-Gonçalves D., Amado F., Duarte J.A., Ferreira R. (2014). Recent insights on the molecular mechanisms and therapeutic approaches for cardiac cachexia. Clin. Biochem..

[48] Attanasio P., Anker S.D., Doehner W., von Haehling S. (2011). Hormonal consequences and prognosis of chronic heart failure. Curr. Opin. Endocrinol. Diabetes Obes..

[49] Krysztofiak H., Wleklik M., Migaj J., Dudek M., Uchmanowicz I., Lisiak M., Kubielas G., Straburzyńska-Migaj E., Lesiak M., Kałużna-Oleksy M. (2020). Cardiac Cachexia: A Well-Known but Challenging Complication of Heart Failure. Clin. Interv. Aging.

[50] Yoshida T., Tabony A.M., Galvez S., Mitch W.E., Higashi Y., Sukhanov S., Delafontaine P. (2013). Molecular mechanisms and signaling pathways of angiotensin II-induced muscle wasting: Potential therapeutic targets for cardiac cachexia. Int. J. Biochem. Cell Biol..

[51] Yoshida T., Delafontaine P. (2015). Mechanisms of Cachexia in Chronic Disease States. Am. J. Med. Sci..

[52] Aukrust P., Ueland T., Lien E., Bendtzen K., Müller F., Andreassen A.K., Nordøy I., Aass H., Espevik T., Simonsen S. (1999). Cytokine network in congestive heart failure secondary to ischemic or idiopathic dilated cardiomyopathy. Am. J. Cardiol..

[53] Loncar G., Springer J., Anker M., Doehner W., Lainscak M. (2016). Cardiac cachexia: Hic et nunc. J. Cachexia Sarcopenia Muscle.

[54] Kistorp C., Faber J., Galatius S., Gustafsson F., Frystyk J., Flyvbjerg A., Hildebrandt P. (2005). Plasma Adiponectin, Body Mass Index, and Mortality in Patients with Chronic Heart Failure. Circulation.

[55] Scherbakov N., Bauer M., Sandek A., Szabó T., Töpper A., Jankowska E.A., Springer J., von Haehling S., Anker S.D., Lainscak M. (2015). Insulin resistance in heart failure: Differences between patients with reduced and preserved left ventricular ejection fraction. Eur. J. Heart Fail..

[56] Korek E., Krauss H., Gibas-Dorna M., Kupsz J., Piątek M., Piątek J. (2013). Fasting and postprandial levels of ghrelin, leptin and insulin in lean, obese and anorexic subjects. Prz. Gastroenterol..

[57] Sanders P.M., Russell S.T., Tisdale M.J. (2005). Angiotensin II directly induces muscle protein catabolism through the ubiquitin–proteasome proteolytic pathway and may play a role in cancer cachexia. Br. J. Cancer.

[58] Hill A., Nesterova E., Lomivorotov V., Efremov S., Goetzenich A., Benstoem C., Zamyatin M., Chourdakis M., Heyland D., Stoppe C. (2018). Current Evidence about Nutrition Support in Cardiac Surgery Patients—What Do We Know?. Nutrients.

[59] Stephens R.S., Shah A.S., Whitman G.J.R. (2013). Lung Injury and Acute Respiratory Distress Syndrome After Cardiac Surgery. Ann. Thorac. Surg..

[60] Ellenberger C., Sologashvili T., Cikirikcioglu M., Verdon G., Diaper J., Cassina T., Licker M. (2017). Risk Factors of Postcardiotomy Ventricular Dysfunction in Moderate-to-high Risk Patients Undergoing Open-heart Surgery. Ann. Card. Anaesth..

[61] Lomivorotov V.V., Efremov S.M., Kirov M.Y., Fominskiy E.V., Karaskov A.M. (2017). Low-Cardiac-Output Syndrome After Cardiac Surgery. J. Cardiothorac. Vasc. Anesth..

[62] Cropsey C., Kennedy J., Han J., Pandharipande P. (2015). Cognitive Dysfunction, Delirium, and Stroke in Cardiac Surgery Patients. Semin. Cardiothorac. Vasc. Anesth..

[63] Mangusan R.F., Hooper V., Denslow S.A., Travis L. (2015). Outcomes Associated with Postoperative Delirium After Cardiac Surgery. Am. J. Crit. Care.

[64] Lebreton G., Tamion F., Coeffier M., Richard V., Bubenheim M., Bessou J.P., Doguet F. (2013). Modulation of mesenteric vasoreactivity and inflammatory response by protein undernutrition in cardiopulmonary bypass. Nutrition.

[65] Ávila N.G.d., Carneiro J.U., Alves F.D., Corrêa I.V.d.S., Vallandro J.P. (2020). Prevalence of Malnutrition and Its Association with Clinical Complications in Hospitalized Cardiac Patients: Retrospective Cohort Study. Int. J. Cardiovasc. Sci..

[66] Karst F.P., Vieira R.M., Barbiero S. (2015). Relationship between adductor pollicis muscle thickness and subjective global assessment in a cardiac intensive care unit. Rev. Bras. De Ter. Intensiv..

[67] Dong B., Wang J., Li P., Li J., Liu M., Zhang H. (2023). The impact of preoperative malnutrition on postoperative delirium: A systematic review and meta-analysis. Perioper. Med..

[68] Drover J.W., Cahill N.E., Kutsogiannis J., Pagliarello G., Wischmeyer P., Wang M., Day A.G., Heyland D.K. (2010). Nutrition Therapy for the Critically Ill Surgical Patient. J. Parenter. Enter. Nutr..

[69] Ookawara S., Kaku Y., Ito K., Kizukuri K., Namikawa A., Nakahara S., Horiuchi Y., Inose N., Miyahara M., Shiina M. (2019). Effects of dietary intake and nutritional status on cerebral oxygenation in patients with chronic kidney disease not undergoing dialysis: A cross-sectional study. PLoS ONE.

[70] Weimann A., Braga M., Carli F., Higashiguchi T., Hübner M., Klek S., Laviano A., Ljungqvist O., Lobo D.N., Martindale R. (2017). ESPEN guideline: Clinical nutrition in surgery. Clin. Nutr..

[71] Almohammadi A.A., Alqarni M.A., Alqaidy M.Y., Ismail S.A., Almabadi R.M. (2023). Impact of the Prognostic Nutritional Index on Postoperative Outcomes in Patients Undergoing Heart Surgery. Cureus.

[72] Kawanishi H., Ida M., Naito Y., Kawaguchi M. (2023). Effects of preoperative nutritional status on disability-free survival after cardiac and thoracic aortic surgery: A prospective observational study. J. Anesth..

[73] Wiberg S., Holmgaard F., Zetterberg H., Nilsson J.-C., Kjaergaard J., Wanscher M., Langkilde A.R., Hassager C., Rasmussen L.S., Blennow K. (2022). Biomarkers of Cerebral Injury for Prediction of Postoperative Cognitive Dysfunction in Patients Undergoing Cardiac Surgery. J. Cardiothorac. Vasc. Anesth..

[74] Yuan S.M., Lin H. (2019). Postoperative Cognitive Dysfunction after Coronary Artery Bypass Grafting. Braz. J. Cardiovasc. Surg..

[75] Laalou F.Z., Carre A.C., Forestier C., Sellal F., Langeron O., Pain L. (2008). Physiopathologie de la dysfonction cognitive postopératoire du sujet âgé: Hypothèses actuelles. J. De Chir..

[76] Cacciatore S., Spadafora L., Bernardi M., Galli M., Betti M., Perone F., Nicolaio G., Marzetti E., Martone A.M., Landi F. (2023). Management of Coronary Artery Disease in Older Adults: Recent Advances and Gaps in Evidence. J. Clin. Med..

[77] Alam A., Hana Z., Jin Z., Suen K.C., Ma D. (2018). Surgery, neuroinflammation and cognitive impairment. EBioMedicine.

[78] Hovens I., Schoemaker R., Van der Zee E., Heineman E., Izaks G., van Leeuwen B. (2012). Thinking through postoperative cognitive dysfunction: How to bridge the gap between clinical and pre-clinical perspectives. Brain Behav. Immun..

[79] Plas M., Rotteveel E., Izaks G.J., Spikman J.M., van der Wal-Huisman H., van Etten B., Absalom A.R., Mourits M.J.E., de Bock G.H., van Leeuwen B.L. (2017). Cognitive decline after major oncological surgery in the elderly. Eur. J. Cancer.

[80] Skvarc D.R., Berk M., Byrne L.K., Dean O.M., Dodd S., Lewis M., Marriott A., Moore E.M., Morris G., Page R.S. (2018). Post-Operative Cognitive Dysfunction: An exploration of the inflammatory hypothesis and novel therapies. Neurosci. Biobehav. Rev..

[81] Cibelli M., Fidalgo A.R., Terrando N., Ma D., Monaco C., Feldmann M., Takata M., Lever I.J., Nanchahal J., Fanselow M.S. (2010). Role of interleukin-1β in postoperative cognitive dysfunction. Ann. Neurol..

[82] Jiang P., Ling Q., Liu H., Tu W. (2015). Intracisternal administration of an interleukin-6 receptor antagonist attenuates surgery-induced cognitive impairment by inhibition of neuroinflammatory responses in aged rats. Exp. Ther. Med..

[83] Hovens I.B., van Leeuwen B.L., Falcao-Salles J., de Haan J.J., Schoemaker R.G. (2021). Enteral enriched nutrition to prevent cognitive dysfunction after surgery; a study in rats. Brain Behav. Immun. Health.

[84] Pathirana A.K., Lokunarangoda N., Ranathunga I., Santharaj W.S., Ekanayake R., Jayawardena R. (2014). Prevalence of hospital malnutrition among cardiac patients: Results from six nutrition screening tools. Springerplus.

[85] Lomivorotov V.V., Efremov S.M., Boboshko V.A., Nikolaev D.A., Vedernikov P.E., Lomivorotov V.N., Karaskov A.M. (2013). Evaluation of nutritional screening tools for patients scheduled for cardiac surgery. Nutrition.

[86] Hill D., Conner M., Clancy F., Moss R., Wilding S., Bristow M., O’Connor D.B. (2022). Stress and eating behaviours in healthy adults: A systematic review and meta-analysis. Health Psychol. Rev..

[87] Nitichai N., Angkatavanich J., Somlaw N., Voravud N., Lertbutsayanukul C. (2019). Validation of the Scored Patient-Generated Subjective Global Assessment (PG-SGA) in Thai Setting and Association with Nutritional Parameters in Cancer Patients. Asian Pac. J. Cancer Prev..

[88] Liu L., Jin J., Wang M., Xu X., Jiang H., Chen Z., Li Y., Gao J., Zhang W. (2023). Association of Pre-procedural Nutritional Indicators with Periprocedural Myocardial Infarction in Patients Undergoing Elective Percutaneous Coronary Intervention. Int. Heart J..

[89] Ikeya Y., Saito Y., Nakai T., Kogawa R., Otsuka N., Wakamatsu Y., Kurokawa S., Ohkubo K., Nagashima K., Okumura Y. (2021). Prognostic importance of the Controlling Nutritional Status (CONUT) score in patients undergoing cardiac resynchronisation therapy. Open Heart.

[90] Weimann A., Braga M., Carli F., Higashiguchi T., Hübner M., Klek S., Laviano A., Ljungqvist O., Lobo D.N., Martindale R.G. (2021). ESPEN practical guideline: Clinical nutrition in surgery. Clin. Nutr..

[91] Li J.-S., Shuang W.-B. (2024). Perioperative nutrition optimization: A review of the current literature †. Front. Nurs..

[92] Singer P., Blaser A.R., Berger M.M., Alhazzani W., Calder P.C., Casaer M.P., Hiesmayr M., Mayer K., Montejo J.C., Pichard C. (2019). ESPEN guideline on clinical nutrition in the intensive care unit. Clin. Nutr..

[93] Sun L.Y., Spence S.D., Benton S., Beanlands R.S., Austin P.C., Bader Eddeen A., Lee D.S. (2022). Age, Not Sex, Modifies the Effect of Frailty on Long-term Outcomes after Cardiac Surgery. Ann. Surg..

